# Supplementary material for: High-performance cryo-temperature ionic thermoelectric liquid cell developed through a eutectic solvent strategy
Source: Nat Commun. 2024 Feb 8;15:1172. doi: 10.1038/s41467-024-45432-7 (PMC10853189; doi:10.1038/s41467-024-45432-7)
Supplement: Supplementary file 1 — Supplementary Information [file 41467_2024_45432_MOESM1_ESM.pdf]

Supporting Information

High-Performance Cryo-Temperature Ionic Thermoelectric Liquid Cell Developed through a Eutectic Solvent Strategy

Shuaihua Wang[1], Yuchen Li[1], Mao Yu[1], Qikai Li[1], Huan Li[1], Yupeng Wang[1], Jiajia Zhang[1], Kang Zhu[1], Weishu Liu[1]*

[1]*Department of Materials Science and Engineering, Southern University of Science and Technology, Shenzhen, Guangdong 518055, China*

These authors contributed equally: Shuaihua Wang, Yuchen Li.

*Corresponding author. Email: liuws@sustech.edu.cn

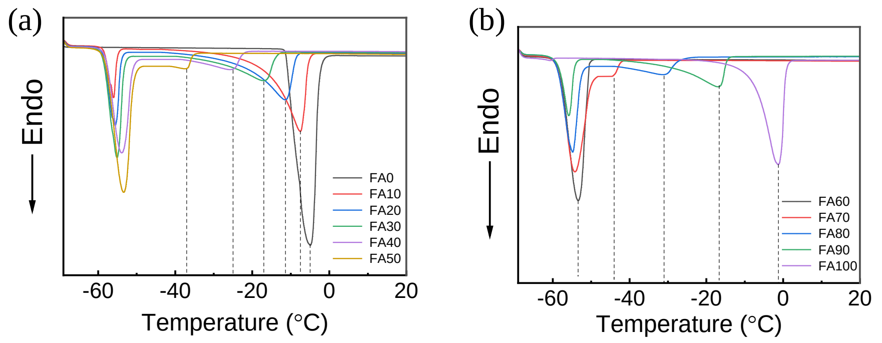

Supplementary Figure 1. The differential scanning calorimetry (DSC) measurement of the ionic solution of H_2O/x FA-0.4M $FeCN^{4-/3-}$-3M GdmCl. a) x = 0 vol.%, 10 vol.%, 20 vol.%, 30 vol.%, 40 vol.%, 50 vol.%, which were referred as FA0, FA10, FA 20, FA30, FA40, FA50. b) x = 60 vol.%, 70 vol.%, 80 vol.%, 90 vol.%, 100 vol.%, which were referred as FA60, FA70, FA 80, FA90, FA100.

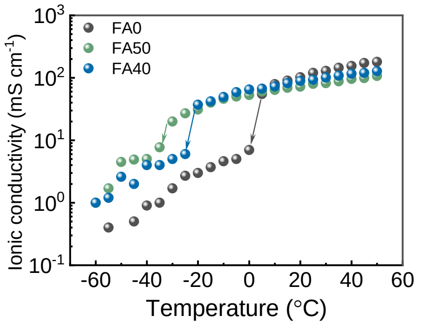

Supplementary Figure 2. The ionic conductivities of the ionic solution (FA0, FA40, and FA50) in a temperature range from 50 to -60°C.

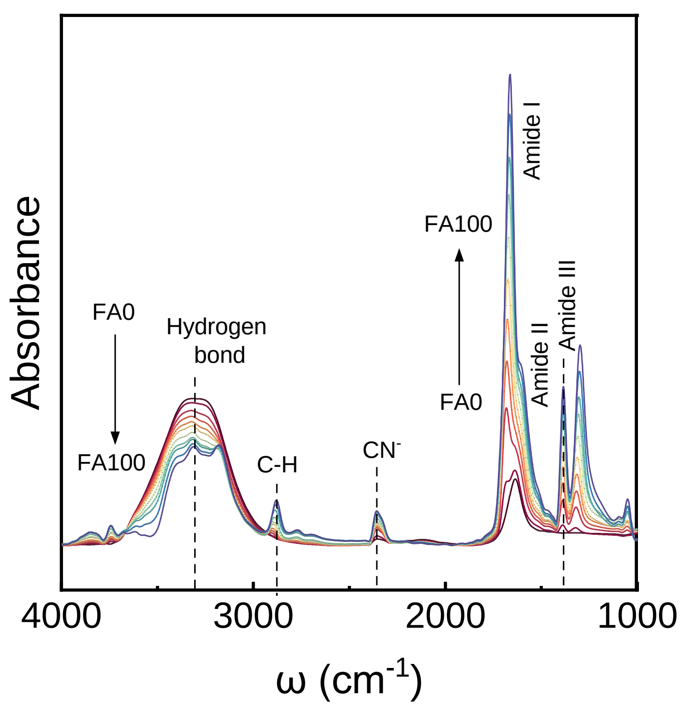

Supplementary Figure 3. Fourier Transform Infrared spectroscopy (FTIR) measurement of the ionic solution of H_2O/*x* FA-$FeCN^{4-/3-}$-GdmCl (*x* = 0–100 vol.% in 10-vol.% increments).

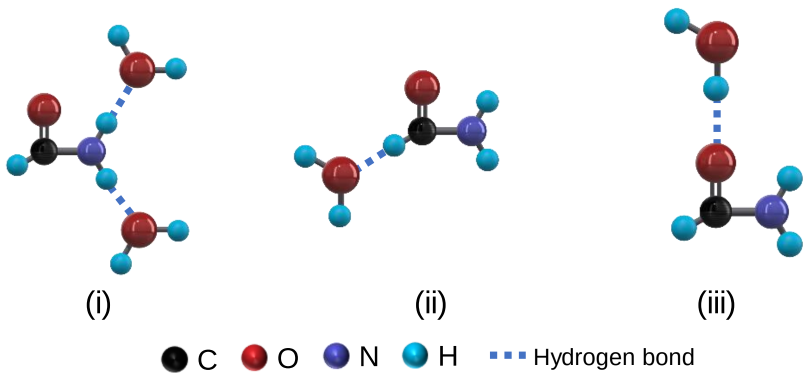

Supplementary Figure 4. Three different forms of hydrogen bond between formamide (FA) and water.

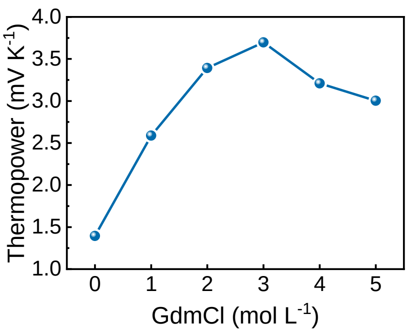

Supplementary Figure 5. Thermopower versus concentration of GdmCl for the i-TE liquid cell of H_2O/*x* FA-*y* $FeCN^{4-/3-}$-*z* GdmCl (*x* = 0 vol.%, *y* = 0.4M, *z* = 0, 1, 2, 3, 4, 5M).

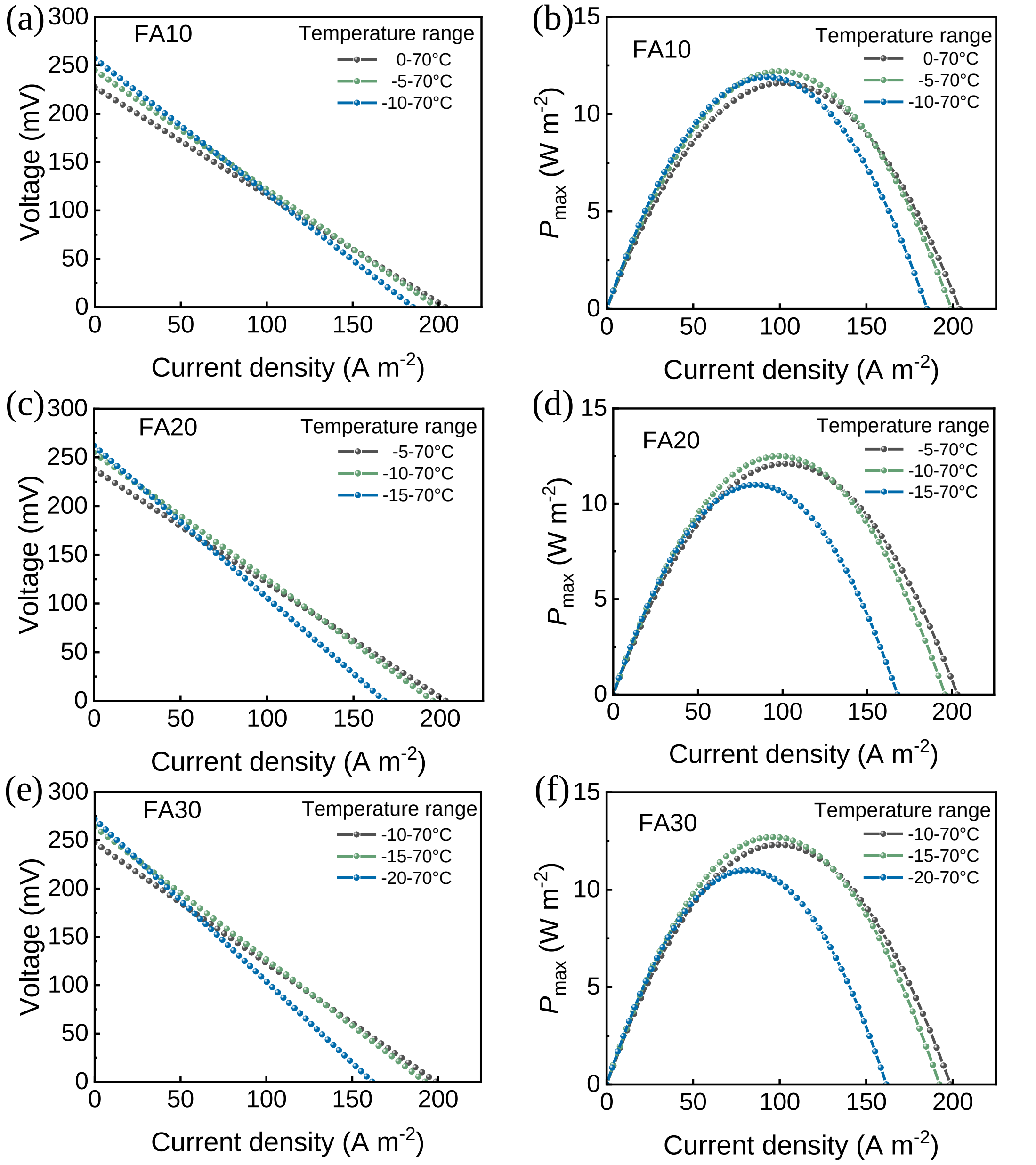

Supplementary Figure 6. Voltage and output power density versus current density for the as-fabricated i-TE liquid cell of H_2O/*x* FA-0.4M $FeCN^{4-/3-}$-3M GdmCl (h = 14mm) under different T_c with a fixed T_h = 70 °C. a) and b) x = 10 vol.%, T_c = 0, -5, and -10°C. c) and d) x = 20 vol.%, T_c = -5, -10, and -15°C. e) and f) x = 30 vol.%, T_c = -10, -15, and -20°C.

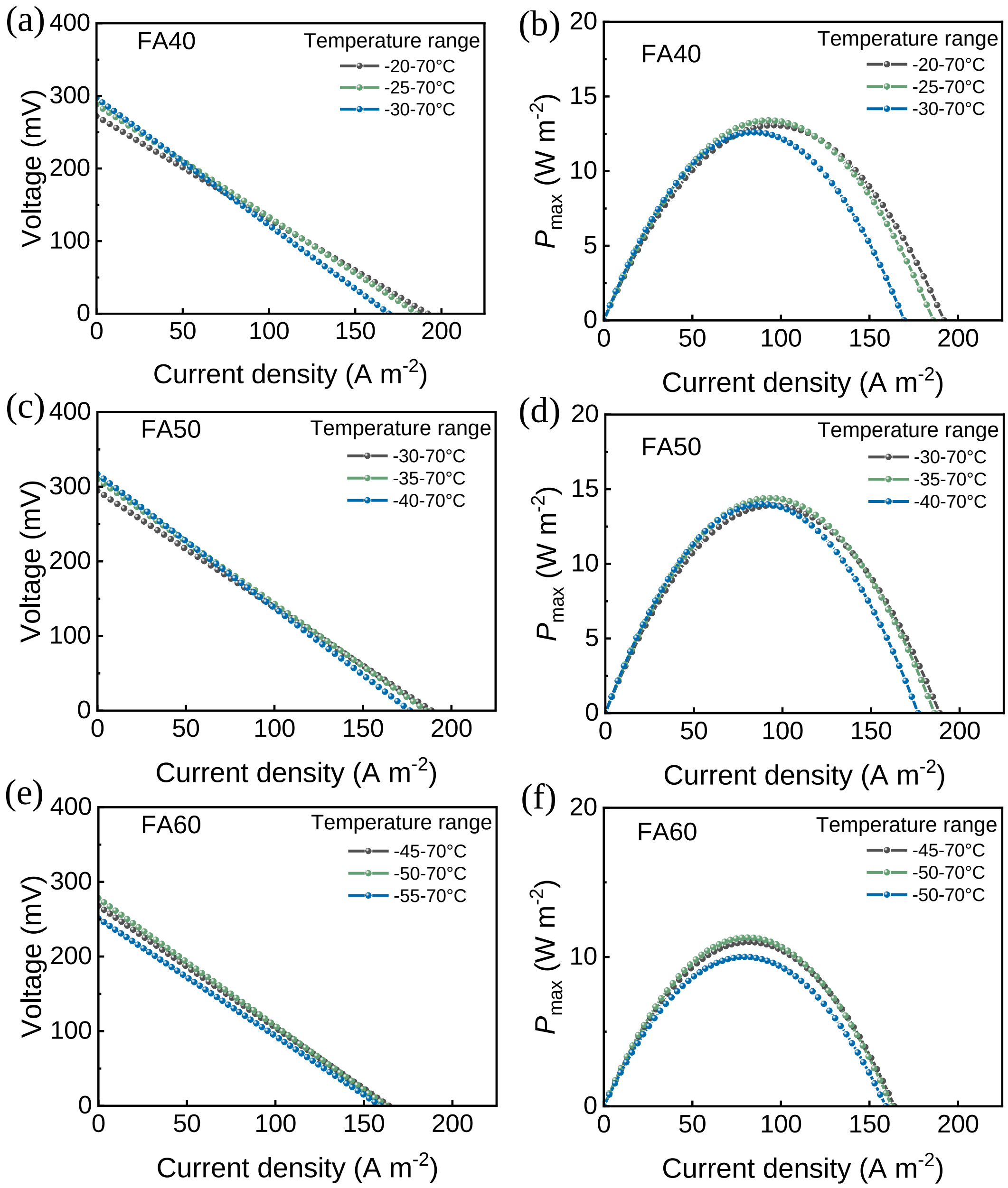

Supplementary Figure 7. Voltage and output power density versus current density for the as-fabricated i-TE liquid cell of H_2O/*x* FA-0.4M $FeCN^{4-/3-}$-3M GdmCl (h = 14mm) under different T_c with a fixed T_h = 70 °C. a) and b) x = 40 vol.%, T_c = -20, -25, and -30°C. c) and d) x = 50 vol.%, T_c = -30, -35, and -40°C. e) and f) x = 60 vol.%, T_c = -45, -50, and -55°C.

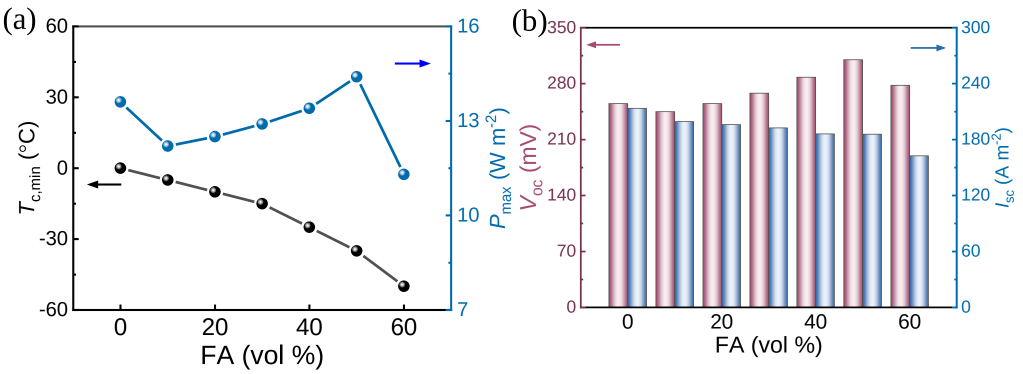

Supplementary Figure 8. The effects of the content of FA on the performance of i-TE liquid cells. a) $T_{c,min}$, P_{max} and b) V_{oc}, and I_{sc} for the i-TE liquid cell of H_2O/*x* FA-0.4M $FeCN^{4-/3-}$-3M GdmCl (*h* = 14mm) working at the corresponding ΔT_{max} (*x* = 0 vol%, 10 vol%, 20 vol%, 30 vol%, 40 vol%, 50 vol%, 60 vol%).

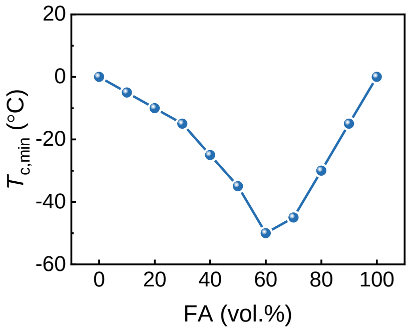

Supplementary Figure 9. $T_{c,min}$ versus the content of FA for the as-fabricated i-TE liquid cell of H_2O/*x* FA-0.4M $FeCN^{4-/3-}$-3M GdmCl (h = 14mm) (x = 0 vol%, 10 vol%, 20 vol%, 30 vol%, 40 vol%, 50 vol%, 60 vol%).

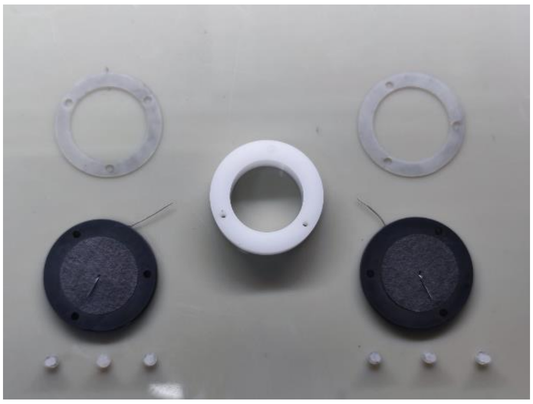

Supplementary Figure 10. The as-fabricated i-TE cell is assembled in laminar structure in consist of two graphite current collectors, two carbon paper electrodes, a thermal separator attached to the cold-side electrode, a cylinder spacer made of polyformaldehyde (commercial sources) that defines the electrode separation gap (*h*), the electrolyte volume at cell center, and two rubber O-rings.

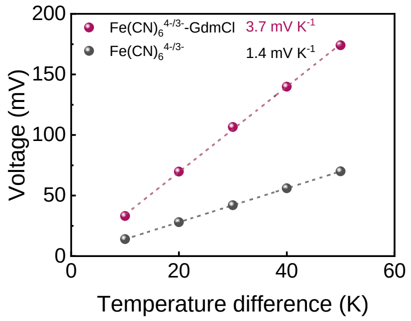

Supplementary Figure 11. The thermopower of 0.4M $K_3Fe(CN)_6$/$K_4Fe(CN)_6$ and 0.4M $K_3Fe(CN)_6$/$K_4Fe(CN)_6$-3M GdmCl measured by the as-fabricated i-TE device in this work.

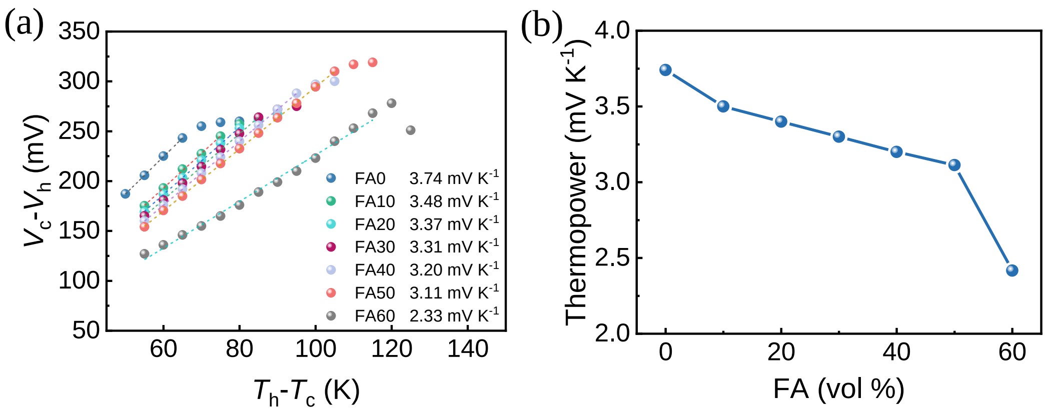

Supplementary Figure 12. a) Dependence of the voltage difference (V_c-V_h) on the temperature difference (T_h-T_c, T_h = 70 °C) for the as-fabricated i-TE liquid cell of H_2O/x FA-0.4M $FeCN^{4-/3-}$ -3M GdmCl (h = 14mm) (x = 0 vol%, 10 vol%, 20 vol%, 30 vol%, 40 vol%, 50 vol%, 60 vol%); b) Thermopower versus the content of FA.

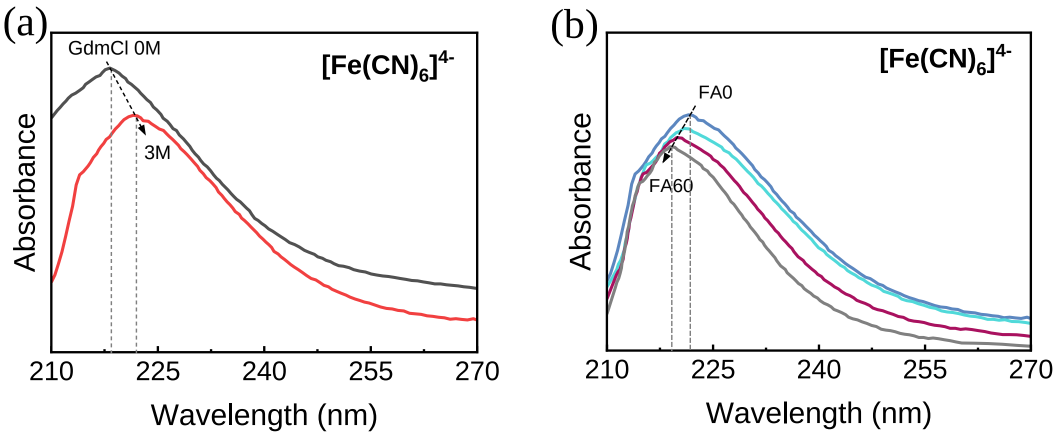

Supplementary Figure 13. UV-Vis spectral shifts and the corresponding absorption peaks for $[FeCN_6]^{4-}$. a) UV-Vis spectra for FA-free $K_4Fe(CN)_6$ solution with and without GdmCl, and b) $K_4Fe(CN)_6$-GdmCl solution with different FA contents.

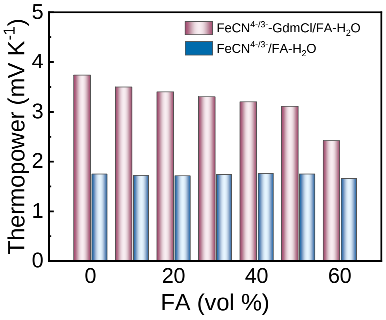

Supplementary Figure 14. Thermopower of 0.4M $FeCN^{4-/3-}$ and 0.4M $FeCN^{4-/3-}$-3M GdmCl with different FA contents.

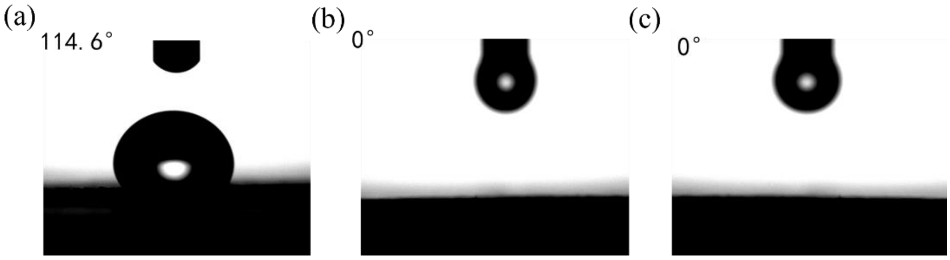

Supplementary Figure 15. Contact angle of the ionic solution (FA50) on a) hydrophobic CP, b) hydrophilic CP, and c) hydrophilic Au@CP electrode is 114.6°, 0° and 0°, which means that the electrolyte has better wettability to the hydrophilic Au@CP electrode.

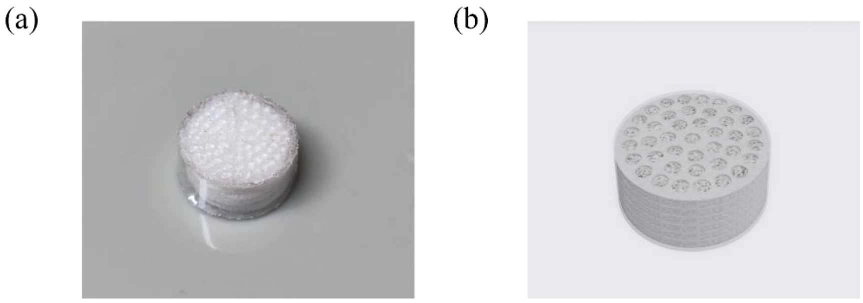

Supplementary Figure 16. a) Entity profile and b) schematic drawing of the thermal insulation separator (TIS) made by cotton fiber laminating. The thickness of TIS is set to half of the electrode separation gap. The density of TIS is 0.15 g cm^{-3}.

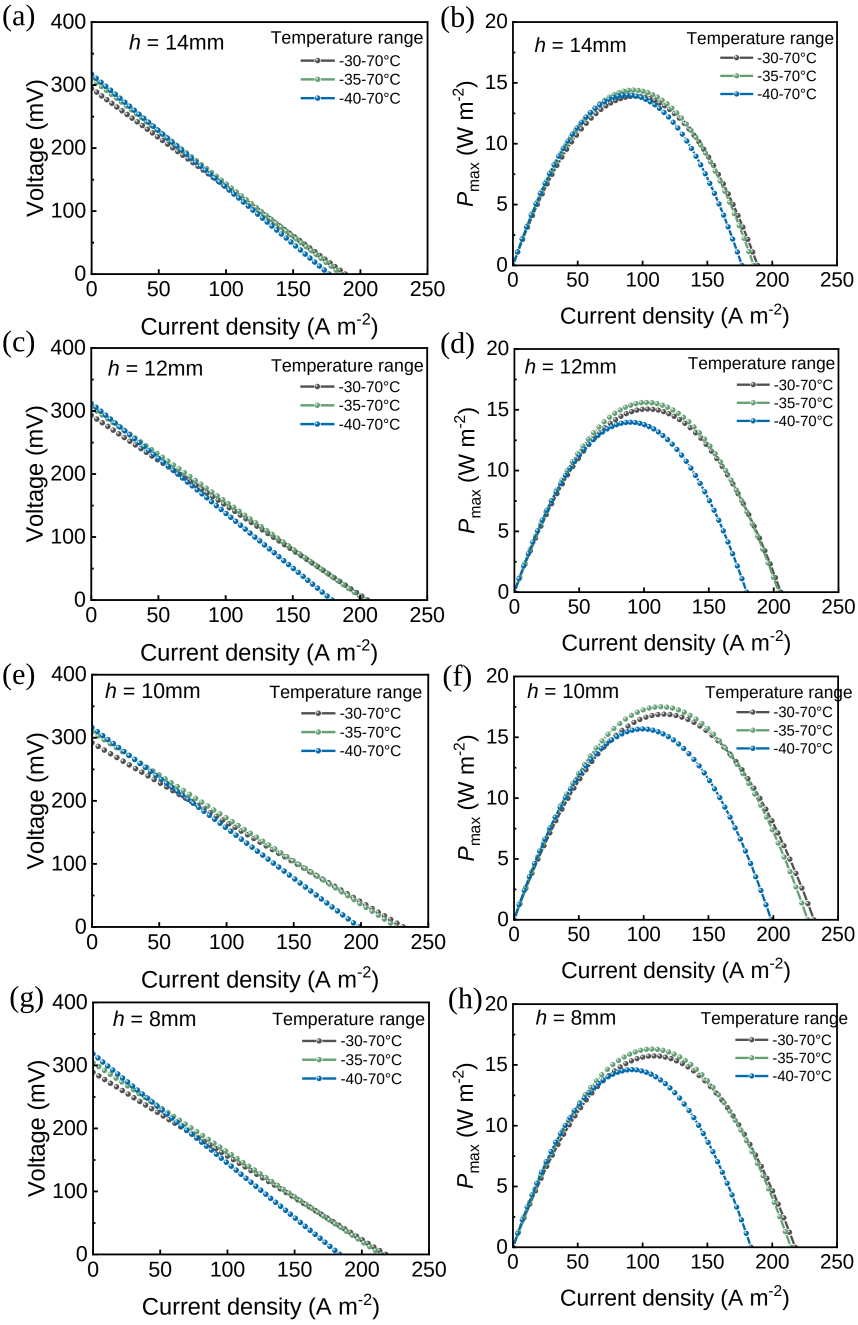

Supplementary Figure 17. Voltage and output power density versus current density for the as-fabricated Hydrophilic Au@CP|H_2O/FA-FeCN$^{4-/3-}$-GdmCl (FA50)|Hydrophilic Au@CP i-TE liquid cell under different T_c with a fixed T_h = 70 °C. a) and b) h = 14mm, T_c = -30, -35, and -40°C. c) and d) h = 12mm, T_c = -30, -35, and -40°C. e) and f) h = 10mm, T_c = -30, -35, and -40°C. g) and h) h = 8mm, T_c = -30, -35, and -40°C.

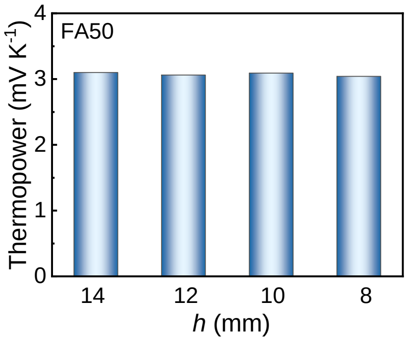

Supplementary Figure 18. Thermopower versus electrode separation gap (*h*) for the as-fabricated Hydrophilic Au@CP|H_2O/FA-FeCN$^{4-/3-}$-GdmCl (FA50)|Hydrophilic Au@CP i-TE liquid cell.

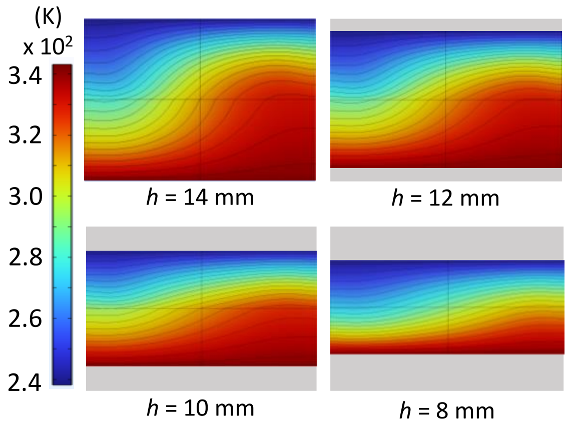

Supplementary Figure 19. The simulated temperature distribution between the electrodes of the FA50-TIS i-TE liquid cell at different electrode separation gaps (h = 14, 12, 10, 8 mm). COMSOL Multiphysics 6.0 with a comprehensive model coupling mass and heat transfer and fluid flow was used to simulate the convection and temperature distributions of the solution in the FA50-TIS cell at T_h = 70°C and T_c = -35°C. The cell model was vertically oriented without the use of forced convection, and the temperature difference was built from the bottom (hot) to the top (cold). This free convection problem was modeled by introducing the Boussinesq buoyancy term into the Brinkman momentum equation, and then coupling the resulting fluid velocity with the "Heat Transfer in Porous Media" interface. In addition, the parameters used in the simulation are summarized in Table S2.

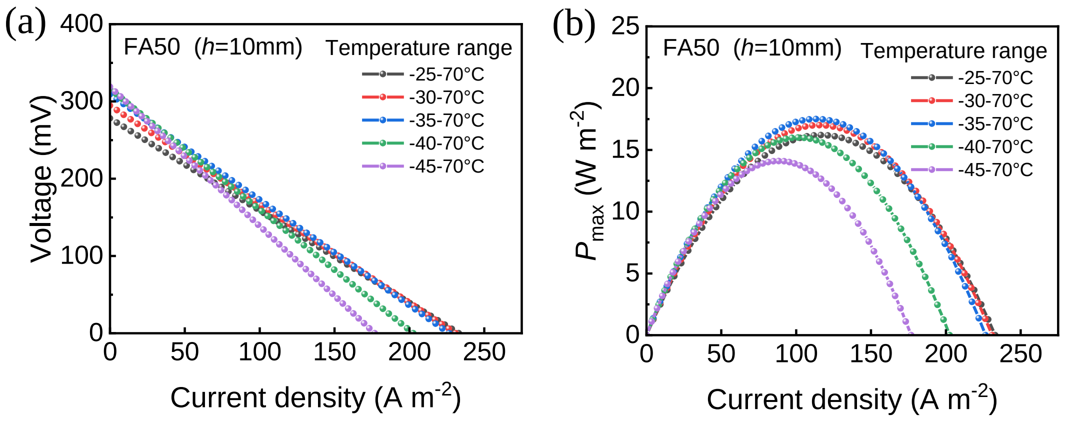

Supplementary Figure 20. a) Voltage and b) output power density versus current density for the optimal sample Hydrophilic Au@CP|H_2O/FA-$FeCN^{4-/3-}$-GdmCl (FA50) (h = 10 mm)|Hydrophilic Au@CP i-TE liquid cell under different T_c (-25, -30, -35, -40, and -45 °C) with a fixed T_h = 70°C.

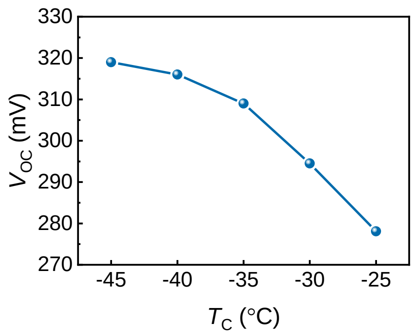

Supplementary Figure 21. V_{oc} versus T_c for the optimal sample Hydrophilic Au@CP|H_2O/50 vol.% FA-$FeCN^{4-/3-}$-GdmCl (FA50) (h = 10 mm)|Hydrophilic Au@CP i-TE liquid cell with a fixed T_h = 70°C.

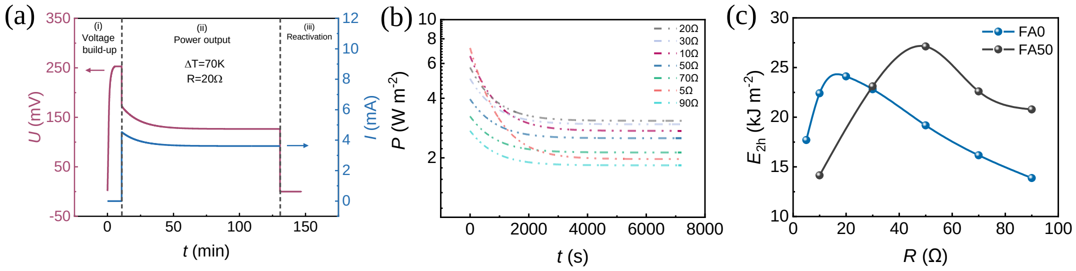

Supplementary Figure 22. Long-term power generation of the *hl*-Au@CP|FA0-TIS|*hl*-Au@CP i-TE liquid cell (h = 10 mm) at T_c = 0°C, T_h = 70°C in i-TE generator working mode: a) Measured voltage and current curves during three working stages with a 20 Ω external resistance. b) Output power density measured over 2 h in stage (ii) using different external resistors. c) Corresponding generated energy density as a function of external resistors, calculated by integrating the output power over time (2 h) shown in (b).

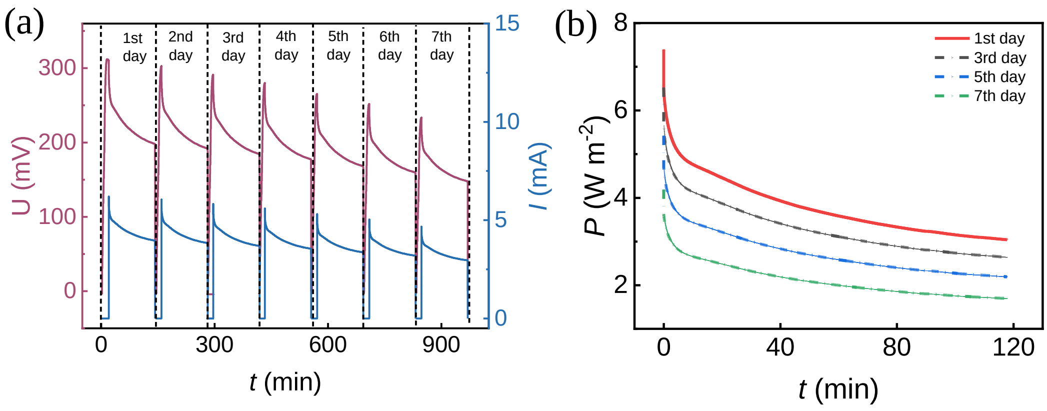

Supplementary Figure 23. Long-term power generation of the *hl*-Au@CP|FA50-TIS|*hl*-Au@CP i-TE liquid cell (h = 10 mm) during one week of continuous cycling (T_h = 70°C, T_c = -35°C). a) The measured voltage and current curves of build-up voltage and power output stages for the first seven days and b) the output power density of the second stage on the first, third, fifth, and seventh day.

(a) (b)

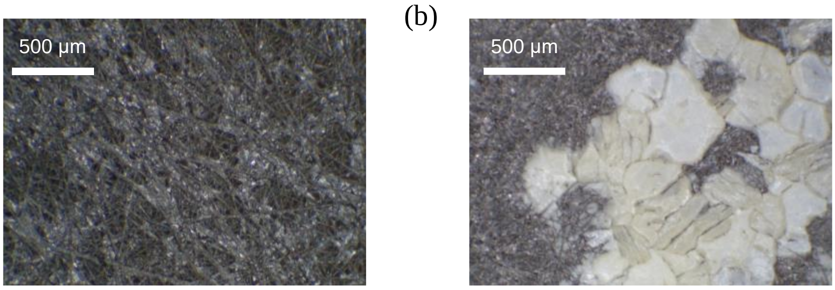

Supplementary Figure 24. Optical microscope image of the electrodes a) before and b) after cycling test at T_h = 70°C. With the one-week continuous cycling test, the electrode surface is gradually covered with a passivation film.

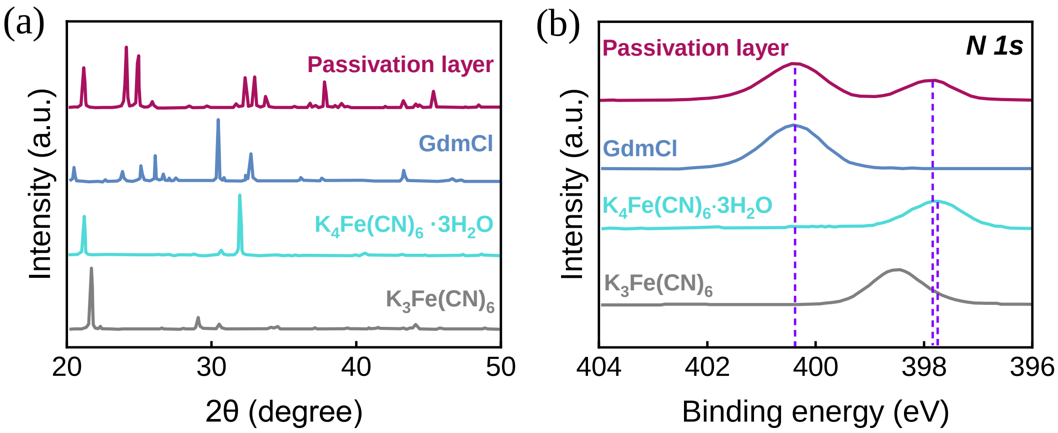

Supplementary Figure 25. a) The XRD spectra, and b) *N 1s* XPS spectra comparison of pure $K_3Fe(CN)_6$, $K_4Fe(CN)_6$, GdmCl powders, and the formed passivation layer, showing their characteristic peaks.

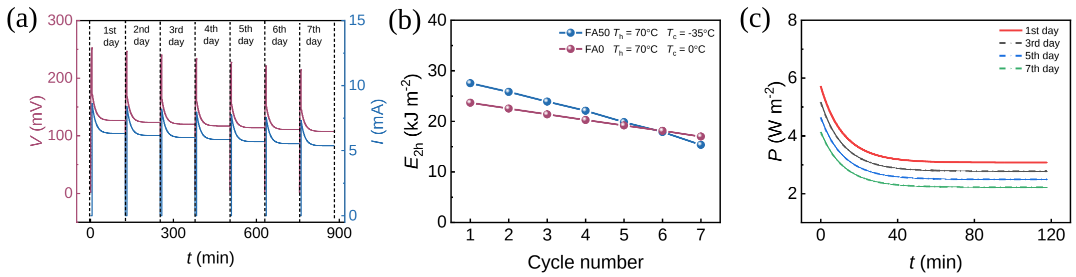

Supplementary Figure 26. Long-term power generation of the *hl*-Au@CP|FA0-TIS|*hl*-Au@CP i-TE liquid cell (h = 10 mm) during one week of continuous cycling (T_h = 70°C, T_c = 0°C). a) The measured voltage and current curves of build-up voltage and power output stages for the first seven days and b) the output power density of the second stage on the first, third, fifth, and seventh day. c) Decay of two-hour energy output E_{2h} of the FA0 (T_h = 70°C, T_c = 0°C) and FA50 (T_h = 70°C, T_c = -35°C) i-TE liquid cells during one week of cyclic service.

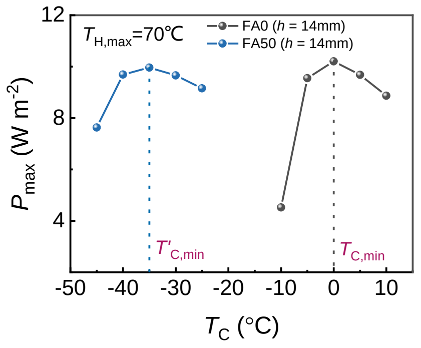

Supplementary Figure 27. Plot of maximum power P_{max} of the *hl*-Au@CP|FA50-TIS|*hl*-Au@CP i-TE liquid cell (*h* = 14 mm) and *hl*-Au@CP|FA0-TIS|*hl*-Au@CP i-TE liquid cell (*h* = 14 mm) versus cold-side temperature T_c (with fixed hot-side temperature T_h = 70°C) after the one-week cycle test.

Supplementary Table 1. Thermal conductivity of electrolyte and electrode.

	Thermal conductivity (W m^{-1} K^{-1})
Graphite current collector	125
hp-CP	1.8
hl-CP	1.8
hl-Au@CP	1.7
H_2O/FA-0.4M $FeCN^{4-/3-}$	1.6
FA50	0.4
FA50 with TIS	0.3

Supplementary Table 2. Calculation parameters for COMSOL simulation.

Parameter	Value
Cell radius	1cm
Hot bottom temperature	343K
Cold top temperature	238K
Solution density	1096.7 kg m^{-3}
Kinematic viscosity	0.6×10^{-6} m^2 s^{-1}
Solution specific heat capacity	4187 J kg^{-1} K^{-1}
Solution thermal conductivity (κ)	0.55 W m^{-1} K^{-1}
Separator porosity	0.3
Separator permeability	2.35m^2
Sediment porosity	0.16
Sediment permeability	1.29m^2
